# Cell Proteins Obtained by Peptic Shaving of Two Phenotypically Different Strains of *Streptococcus thermophilus* as a Source of Anti-Inflammatory Peptides

**DOI:** 10.3390/nu14224777

**Published:** 2022-11-11

**Authors:** Rania Allouche, Magali Genay, Annie Dary-Mourot, Zeeshan Hafeez, Laurent Miclo

**Affiliations:** CALBINOTOX, Université de Lorraine, F-54000 Nancy, France

**Keywords:** *Streptococcus thermophilus*, surface proteins, pepsin, trypsin, inflammatory mediators

## Abstract

*Streptococcus thermophilus*, a food grade bacterium, is extensively used in the manufacture of fermented products such as yogurt and cheeses. It has been shown that *S. thermophilus* strains exhibited varying anti-inflammatory activities in vitro. Our previous study displayed that this activity could be partially due to peptide(s) generated by trypsin hydrolysis of the surface proteins of *S. thermophilus* LMD-9. Surface protease PrtS could be the source of these peptides during gastrointestinal digestion. Therefore, peptide hydrolysates were obtained by shaving two phenotypically distinct strains of *S. thermophilus* (LMD-9 PrtS^+^ and CNRZ-21N PrtS^−^) with pepsin, a gastric protease, followed or not by trypsinolysis. The peptide hydrolysates of both strains exhibited anti-inflammatory action through the modulation of pro-inflammatory mediators in LPS-stimulated THP-1 macrophages (COX-2, Pro-IL-1β, IL-1β, and IL-8) and LPS-stimulated HT-29 cells (IL-8). Therefore, peptides released from either PrtS^+^ or PrtS^−^ strains in the gastrointestinal tract during digestion of a product containing this bacterium may display anti-inflammatory effects and reduce the risk of inflammation-related chronic diseases.

## 1. Introduction

*Streptococcus thermophilus* is a food grade bacterium [1], as it is the only species of *Streptococcus* genus considered as generally recognized as safe (GRAS) by the FDA and has gained qualified presumption of safety (QPS) status from EFSA. In the dairy industry, it is the second most employed lactic acid bacteria after *Lactococcus lactis* to produce yogurt and cheeses [2,3]. In addition to improving the organoleptic properties of products, *S. thermophilus* also rapidly acidifies the milk using its surface proteolytic system. Some strains of *S. thermophilus* display three distinct proteases at their cell surface, namely, the cell-envelope proteinase PrtS, house-keeping protease HtrA, and protease SepM [4]. Nevertheless, among these proteases, PrtS is the only one that distinguishes *S. thermophilus* strains into two phenotypes, i.e. PrtS^+^ phenotype (strains displaying PrtS activity) and the PrtS^−^ phenotype (strains lacking PrtS activity) [4,5,6,7]. PrtS is the key surface protease in *S. thermophilus*, which is responsible not only for the degradation of milk caseins but is also capable of producing diverse peptides with different bioactivities from milk proteins [8,9].

In recent years, interest in the valorization of food protein-derived bioactive peptides carrying various health-promoting properties has increased. Such a bioactive peptide, encrypted in the native protein sequence, is functionally inactive. To exert its specific bioactivity, it should be released by enzymatic hydrolysis in vitro or during food processing/fermentation or gastrointestinal digestion [10]. Indeed, not only the digestion of proteins from food, but also the endogenous proteins by digestive proteases release peptides with several biological activities [11,12,13]. Numerous food protein-derived peptides exhibiting biological activities such as antihypertensive, antimicrobial, antithrombotic, antioxidant, hypocholesterolemic, immunomodulatory, opioid, and mineral binding activities have already been reported [14,15].

Aside from being able to generate bioactive peptides from milk proteins, live or heat-inactivated cells of *S. thermophilus* also displayed anti-inflammatory activities in both in vitro and in vivo studies [16,17,18]. Junjua et al. reported the anti-inflammatory activity of *S. thermophilus* strains (both PrtS^+^ and PrtS^−^) as they decreased IL-8 secretion in HT-29 cell line and displayed a high IL-10/IL-12 ratio in peripheral blood mononuclear cells (PBMC) [19], although the mechanism of this activity was not explained by the authors. Few studies have shown that bacterial surface proteins can exhibit an anti-inflammatory activity, e.g., *Lactobacillus acidophilus* surface-layer protein (Slps) decreased IL-8 secretion in *Salmonella Typhimurium*-stimulated Caco-2 cells [20] whereas *Propionibacterium freudenreichii* CIRM-BIA 129 surface-layer protein B (SlpB) decreased IL-8 expression in HT-29 cells stimulated by LPS [21]. Generally, different types of proteins are present at the surface of Gram-positive bacteria: integral membrane proteins, lipoproteins as well as proteins possessing the LPXTG motif that are covalently attached to membrane lipids and peptidoglycan, respectively, moonlighting proteins, and non-covalently bound proteins [22,23]. *S. thermophilus* is a Gram-positive bacteria and as an integral part of the food bolus, proteins of this bacterium are added to those of other food components as well as to endogenous secretions of the digestive tract and could be a source of bioactive peptides.

In our previous study, it was shown that a peptide hydrolysate generated by tryptic shaving of surface proteins of *S. thermophilus* LMD-9 (PrtS^+^ strain) exhibited anti-inflammatory activities in THP-1 macrophages as well as in HT-29 cells [24]. In addition, mass spectrometry analysis revealed that the surface protease PrtS was the second most peptide-generating protein in our conditions. In line with these findings, it was proposed that the highlighted anti-inflammatory activities could be partially due to peptide(s) generated from PrtS [24]. However, it is important to note that PrtS^−^ strains that lack PrtS also displayed anti-inflammatory activity in in vitro cell models [19]. Hence, the hypothesis that peptides from other surface proteins can also take part in the overall anti-inflammatory activity of this bacterium cannot be ruled out. Moreover, since pepsin is the first enzyme encountered in gastrointestinal tract (GIT), the question as to whether this enzyme is able to release anti-inflammatory fragments after the shaving of *S. thermophilus* has arisen.

Therefore, the aim of the present study was to determine whether shaving of two phenotypically distinct strains of *S. thermophilus* (LMD-9 PrtS^+^ and CNRZ-21N PrtS^−^) with pepsin followed or not by trypsin hydrolysis is able to generate anti-inflammatory peptide(s) and to evaluate the overall contribution of PrtS to these activities. The anti-inflammatory activity of peptide hydrolysates, characterized by LC-MS/MS, was studied in two cell models, i.e. intestinal epithelial HT-29 cells and human THP-1 macrophages.

## 2. Materials and Methods

### 2.1. Cells, Chemicals, and Materials

Human monocytic leukemia cell line THP-1 and human colon adenocarcinoma cell line HT-29 were purchased from the European Collection of Authenticated Cell Cultures (ECACC, Sigma-Aldrich, Saint Quentin Fallavier, France). Pepsin from porcine gastric mucosa (EC 3.4.23.1), sequencing grade trypsin (EC 3.4.21.4) from bovine pancreas, phorbol 12-myristate 13-acetate (PMA), lipopolysaccharide (LPS) from *Escherichia coli* O111:B4, dexamethasone (DEX), Roswell Park Memorial Institute (RPMI) 1640 culture medium, Dulbecco’s phosphate-buffered saline (PBS), penicillin/streptomycin, sodium pyruvate, fetal bovine serum (FBS) for THP-1 cells, protease, and phosphatase inhibitor cocktail, cytotoxicity detection kit, and the β-actin antibody used as the reference were purchased from Sigma-Aldrich (Saint Quentin Fallavier, France). Trypsin/ethylenediaminetetraacetic acid (EDTA), McCoy’s 5A medium, and FBS for HT-29 cells were bought from Gibco (Villebon-sur-Yvette, France). ELISA kits for tumor necrosis factor (TNF-α), interleukin-1β (IL-1β), and interleukin-8 (IL-8) were obtained from Thermo Fisher Scientific (Villebon-sur-Yvette, France). The radio-immunoprecipitation assay (RIPA) lysis buffer and BCA protein quantification kit were obtained from Thermo Fisher Scientific (Illkirch-Graffenstaden, France). IL-1β and COX-2 antibodies were purchased from R&D systems (Rennes, France).

### 2.2. Bacterial Strains and Growth Conditions

*Streptococcus thermophilus* LMD-9 (ATCC BAA-491; PrtS^+^) was purchased from ATCC (American Type Culture Collection, Manassas, VA, USA) whereas *S. thermophilus* CNRZ-21N (PrtS^−^) was isolated in our laboratory from a yogurt. Both strains were conserved in 10% (*w*/*v*) sterile reconstituted skim milk at −20 °C. The strains were initially precultured according to Allouche et al. (2022) [24] by inoculating sterile reconstituted skim milk with 1% inoculum, which was then incubated overnight at 42 °C. M17 broth supplemented with 2% of lactose (LM17) [25] was then inoculated at 1% with the preculture and incubated at 42 °C. The bacterial growth was followed by determining the optical density at 650 nm (OD_650nm_).

### 2.3. Enzymatic Shaving of Surface Proteins

Surface proteins of *S. thermophilus* LMD-9 and CNRZ-21N strains were shaved as described by Lecomte et al. (2014) [26] and adapted according to Allouche et al. (2022) [24]. Shortly, cells of both strains were grown to an OD_650nm_ of 4 in LM17 medium and then concentrated to an OD_650nm_ of 30, as described by Allouche et al. (2022) [24]. For the pepsin shaving of surface proteins, 1 mL of the concentrated cell suspension (resuspended in PBS 10 mM at pH 2.0) was put in contact with 10 µg of pepsin for 1 h at 37 °C with shaking at 180 rpm. To recover the supernatant of the pepsinolysis medium, the solution was centrifuged at 10,000× *g* for 10 min at 20 °C and filtered through 0.45 μm filters (Millipore, Molsheim, France). Afterward, a part of the supernatant obtained after pepsinolysis was heated at 95 °C for 5 min to inactivate pepsin and stored until mass spectrometry analysis. The other part, after neutralization with 20 μL of 1 M NaOH, was hydrolyzed with 2 μg of trypsin for 15 h at 37 °C with shaking (100 rpm). Tryptic hydrolysis was stopped under the same heating conditions that were applied to pepsin. For each strain, part of the samples was conserved at −20 °C until mass spectrometry (LC-MS/MS) analysis and the other part was freeze-dried for evaluation of the anti-inflammatory activity. The negative controls without proteases were carried out under similar conditions for each strain.

### 2.4. LC-MS/MS Analysis

The peptides obtained after shaving and the subsequent proteolysis of surface proteins of both strains were characterized by LC-MS/MS analysis, as described previously by Allouche et al. (2022) [24]. To identify the peptides, LMD-9 and CNRZ-21N protein fasta files were used. The interpretation of results was performed against the Uniprot database https://www.uniprot.org/ (accessed on 15 November 2021) and loctree3 https://rostlab.org/services/loctree3/ (accessed on 15 November 2021).

### 2.5. Cell Lines, Culture Conditions, Toxicity, Anti-Inflammatory Assay, and ELISA Analysis

The two cell lines, HT-29 cells and THP-1 macrophages, were used to evaluate the capacity of hydrolysates from the *S. thermophilus* LMD-9 and CNRZ-21N strains to reduce the secretion of pro-inflammatory cytokines. Both types of cells were grown and induced with LPS, as previously described by Allouche et al. (2022) [24]. The positive control (PC) cells were treated only with LPS while non-treated cells were used as the negative control (NC). After co-incubation with peptide hydrolysate, the IL-8 amount secreted by HT-29 cells, and of IL-1β, TNF-α, and IL-8 by THP-1 macrophages was quantified. THP-1 macrophages or HT-29 cells incubated with culture medium alone were used as the control in each experimental trial. For each strain, experiments were repeated three times and every time, three technical replicates were performed. Toxicity of the peptide hydrolysates toward cells was also assessed according to Allouche et al. (2022) [24].

### 2.6. Western Blot Analysis

Western blot analyses were carried out according to a previously described method [24]. Shortly, total proteins from the treated THP-1 macrophage were extracted using RIPA lysis buffer containing Halt Protease and Phosphatase Inhibitor Cocktail. The BCA protein assay kit was used to determine the protein concentration. Fifteen µg protein samples were separated by 10% SDS-polyacrylamide gel electrophoresis (PAGE) and transferred onto a nitrocellulose membrane (Bio-Rad). After blocking with 5% skimmed milk powder for 1 h at room temperature, the membranes were treated with primary antibodies (1:1000) against IL-1β, COX-2, and β-actin at 4 °C for 15 h. Following washing with TBST (Tris-buffered saline Tween 20), the membranes were reacted with horse radish peroxidase (HRP) conjugated secondary antibodies (1:3000) for 1 h at room temperature, as recommended by the supplier (R&D systems, Rennes, France). The detection of proteins was conducted using the ECL Western Blotting Detection Reagent (GE Healthcare, Buc, France). Chemiluminescent signals were analyzed with Image Lab software (Bio-Rad, Roanne, France). β-actin was used to normalize the Pro-IL-1β and COX-2 bands. All results were represented as the mean ± standard error of mean (SEM) from three experiments performed independently (N = 3).

### 2.7. Statistical Analyses

The means were compared by one-way analysis of variance (ANOVA) and Dunnett’s test was performed to compare the statistical differences between samples. Differences were considered significant at a *p*-value of <0.05. The GraphPad Prism 9.0.2 (GraphPad Software, San Diego, CA, USA) was used to conduct all statistical analyses.

## 3. Results

### 3.1. Analysis of Peptides Recovered after Proteolytic Shaving of S. thermophilus LMD-9 and CNRZ-21N

The peptides from the surface proteins of the *S. thermophilus* LMD-9 and CNRZ-21N strains were obtained by two shaving approaches using digestive enzymes: (1) shaving with pepsin alone; and (2) shaving with pepsin followed by trypsinolysis. The obtained peptides were analyzed by mass spectrometry and are reported in Table 1 and Table 2. About 141 and 122 proteins were identified by the LC-MS/MS analysis of peptides generated after the peptic shaving of *S. thermophilus* LMD-9 and CNRZ-21N, respectively. Only proteins generating more than eight specific sequences were retained for further analysis, resulting in 27 and 18 proteins from LMD-9 and CNRZ-21N, respectively (Table 1). Among the identified surface proteins, the modular protein (2′:3′-cyclic nucleotide 2′-phosphodiesterase) and the PrtS protein were the major peptide generating cell surface proteins in the LMD-9 strain with 205 and 343 specific identified sequences, respectively. In contrast, these proteins were absent in the CNRZ-21N strain, where the peptic shaving caused the release of seventeen specific sequences from two other proteins, i.e. phosphocarrier HPr (histidine-containing protein) and the large subunit of the chaperonin GroEL.

In the case of the second shaving approach, i.e. peptic shaving followed by trypsinolysis of surface proteins of LMD-9 and CNRZ-21N strains, about 95 and 170 proteins were identified, respectively. Only proteins generating more than eight specific sequences were kept for further analysis, resulting in 24 and 19 proteins for LMD-9 and CNRZ-21N, respectively (Table 2).

Regarding the major proteins identified as surface proteins in the LMD-9 strain after tryptic hydrolysis of the peptides generated by pepsin shaving, the modular protein (2′:3′-cyclic nucleotide 2′-phosphodiesterase) and the PrtS protein, which remained the predominant cell surface proteins as 152 and 190 specific sequences, respectively, were identified. In contrast to the LMD-9 strain, these proteins were absent in the CNRZ-21N strain. The pepsin shaving of the CNRZ-21N strain followed by trypsinolysis of peptides resulted in the generation of twenty-one specific sequences from ribosomal protein L24 (BL23) and 19 from two other proteins, i.e. phosphocarrier HPr (histidine-containing protein) and serine endoprotease (protease Do).

### 3.2. Anti-Inflammatory Effect on THP-1 Macrophages of Peptide Hydrolysates

**Figure 1:**
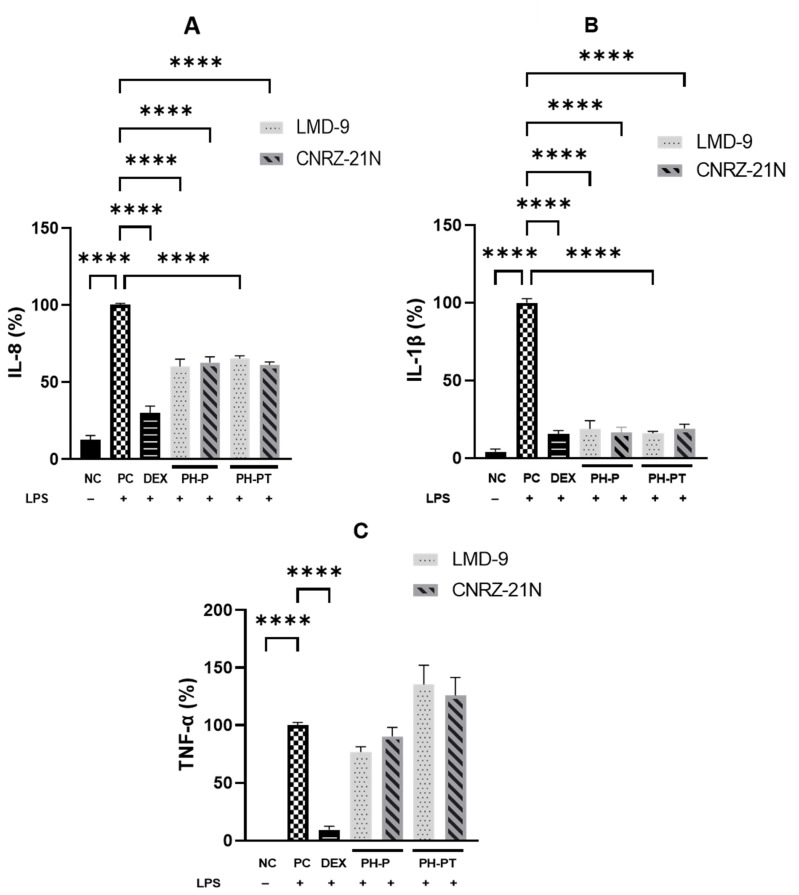
Anti-inflammatory activity highlighted in vitro of hydrolysates recovered after peptic shaving (PH-P), or after peptic shaving followed by trypsinolysis (PH-PT) of *S. thermophilus* LMD-9 and CNRZ-21N. The effect of peptide hydrolysates on secretion of IL-8 (**A**), IL-1β (**B**), and TNF-α (**C**) cytokines in LPS-stimulated THP-1 macrophages. Three hour co-incubation of THP-1 macrophages was performed either with LPS (50 ng/mL) alone or with LPS and (i) PH-P (3 mg/mL) or (ii) PH-PT (3 mg/mL) or (iii) dexamethasone (DEX, 25 µM). After 3 h, the TNF-α, IL-1β, and IL-8 secretion levels were quantified in the cell culture medium by ELISA. The negative control (NC) represents the incubation of untreated cells. IL-8 (%), TNF-α (%), and IL-1β (%) are the percentages of IL-8, TNF-α, or IL-1β, respectively, secreted by cells relative to their production by only LPS-treated cells (PC). All data are represented as the means ± SEM of three experiments performed independently (N = 3). **** *p* < 0.0001.

The anti-inflammatory activity of peptide hydrolysates of *S. thermophilus* LMD-9 and CNRZ-21N strains obtained either with peptic shaving (PH-P) or with peptic shaving followed by trypsin hydrolysis (PH-PT) was first explored in human THP-1 macrophages. Following differentiation, THP-1 macrophages were treated for 3 h only with LPS, an inflammatory activator, as a positive control (PC), or with LPS and DEX, a synthetic glucocorticoid, or LPS and PH-P or PH-PT at 3.0 mg/mL. The pro-inflammatory cytokines (IL1-β, IL-8, and TNF-α) were secreted in significantly higher quantities by LPS-induced THP-1 cells compared to non-induced cells used as a negative control (NC) (*p* < 0.0001, Figure 1). In contrast, the release of IL-1β, IL-8 and TNF-α was potently suppressed by 84.5%, 70.1%, and 90.6% (*p* < 0.0001, Figure 1), respectively, by DEX at 25 µM compared to PC. Likewise, the secretion levels of some pro-inflammatory cytokines were also modified upon the co-treatment of cells with LPS and PH-P or LPS and PH-PT. The secretion of IL-1β by LPS-stimulated THP-1 macrophages was significantly decreased by PH-P or PH-PT treatment. The secretion level of IL-1β was decreased by 81.1% and 83.4% compared to PC for PH-P of LMD-9 and CNRZ-21N strains, respectively (*p* < 0.0001, Figure 1B).

**Figure 2:**
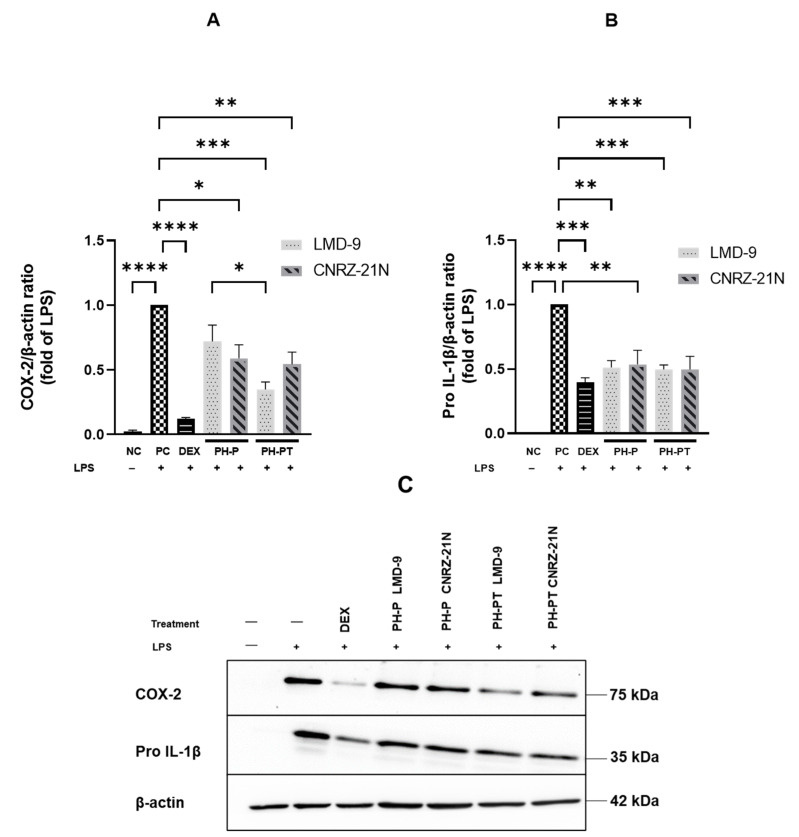
The effects of hydrolysates recovered after peptic shaving (PH-P) or peptic shaving followed by trypsinolysis (PH-PT) of the LMD-9 and CNRZ-21N strains on the protein expression of COX-2 (**A**,**C**) and Pro-IL-1β (**B**,**C**) in the LPS-stimulated THP-1 macrophages. Three hour co-incubation of the THP-1 macrophages was performed either with LPS (50 ng/mL) alone, or with LPS and PH-P (3 mg/mL) or LPS and PH-PT (3 mg/mL) or LPS and dexamethasone (DEX, 25 µM). Protein content (15 µg) of each sample was loaded on 10% SDS-PAGE. The band intensities were determined by image Lab software and the results were calculated as a relative intensity to the β-actin. All data are expressed as the means ± SEM of three experiments performed independently (N = 3). * *p* < 0.05, ** *p* < 0.01, *** *p* < 0.001, **** *p* < 0.0001.

Similarly, PH-PT at 3 mg/mL also showed the same trend of inhibition of IL-1β secretion, which was reduced by 84.1% and 81.0% relative to PC for the LMD-9 and CNRZ-21N strains, respectively (Figure 1B). Interestingly, no significant difference in the secretion levels of IL-1β was observed between the cells treated with DEX at 25 µM and those treated with PH-P or PH-PT at 3 mg/mL. Regarding IL-8 secretion, reduction in the secretion of this cytokine relative to PC (Figure 1A) was statistically significant upon treatment of LPS-induced THP-1 macrophages with PH-P or PH-PT. The PH-P at 3.0 mg/mL from both LMD-9 and CNRZ-21N strains inhibited the secretion of IL-8 by 39.8% and 37.3%, respectively (*p* < 0.0001, Figure 1A). Moreover, PH-PT at 3.0 mg/mL from both the LMD-9 and CNRZ-21N strains suppressed the secretion of IL-8 by 34.7% and 39%, respectively (*p* < 0.0001, Figure 1A). It is also important to note that the secretion of the TNF-α cytokine remained unaffected regardless of the hydrolysate treatment (PH-P or PH-PT) at the same concentrations as those tested for IL-8 and IL-1β secretion (Figure 1C). PH-P and PH-PT were not toxic for the cells at the tested concentrations, since the overall viability of THP-1 macrophages after 3 h contact with these peptide hydrolysates was greater than 98%. The effect of peptide hydrolysates PH-P or PH-PT on the expression of proteins related to inflammation, i.e. Pro-IL-1β and cyclooxygenase-2 (COX-2) was also evaluated by Western blot analysis. The results revealed that the production of these proteins significantly increased in PC cells after 3 h of incubation (*p* < 0.0001; Figure 2). On the other hand, DEX at 25 µM upon co-incubation with THP-1 macrophages stimulated by LPS significantly decreased the COX-2 expression, bringing it back to the basal level of expression. Contrary to PH-P from the LMD-9 strain, PH-P from CNRZ-21N at 3 mg/mL significantly decreased the expression of COX-2 by 41% (*p* < 0.05, Figure 2A) upon co-incubation with LPS-stimulated macrophages. However, the co-treatment of cells with LPS and 3 mg/mL of PH-PT from both the LMD-9 or CNRZ-21N strains reduced the COX-2 expression by 65.1% and 45.6%, respectively (*p* < 0.01, *p* < 0.001) (Figure 2A).

It is interesting to highlight that a significant difference in the decrease of COX-2 expression was observed between cells treated with PH-P or PH-PT obtained from the LMD-9 strain. Like COX-2, the co-treatment of cells with LPS and 3 mg/mL of PH-P from the LMD-9 or CNRZ-21N strains also significantly reduced the expression of Pro-IL-1β protein by 48.8% and 46.5%, respectively (*p* < 0.01; Figure 2B). Pro-IL-1β protein expression was reduced by 50.3% in cells treated with PH-PT of either the LMD-9 or CNRZ-21N strains (*p* < 0.001; Figure 2B).

### 3.3. Anti-Inflammatory Effect on HT-29 Cells of Peptide Hydrolysates

**Figure 3:**
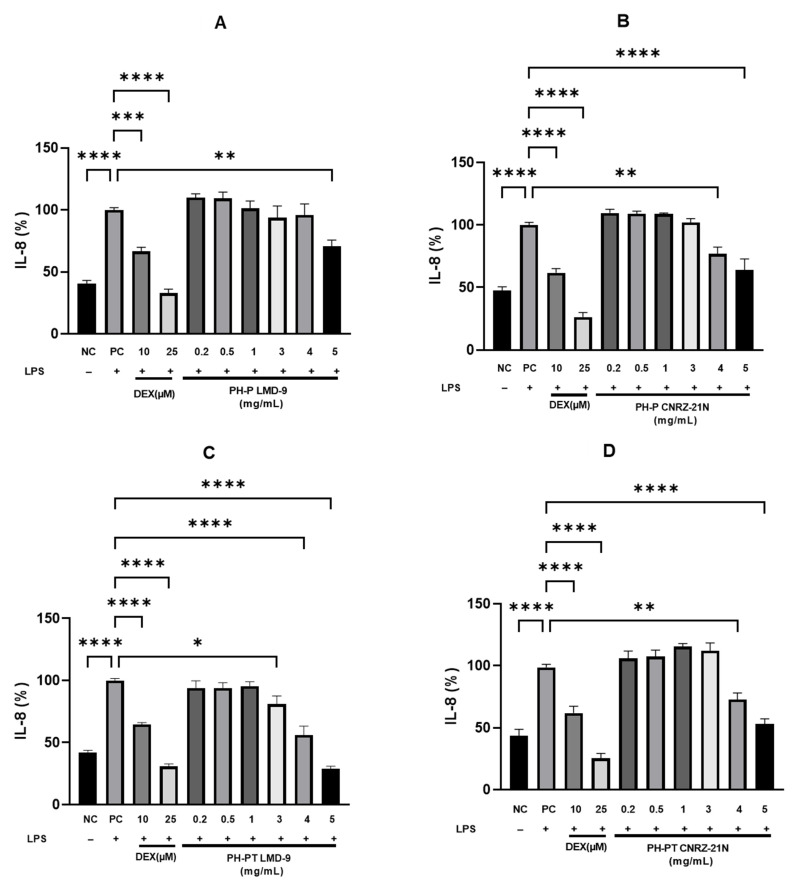
Anti-inflammatory activity highlighted in vitro of hydrolysates recovered after peptic shaving (PH-P) or peptic shaving followed by trypsinolysis (PH-PT) of *S. thermophilus* LMD-9 or CNRZ-21N. The effect of PH-P of LMD-9 (**A**), CNRZ-21N (**B**), and of PH-PT of LMD-9 (**C**), CNRZ-21N (**D**) on the IL-8 secretion in LPS-induced HT-29 cells. Three hour co-incubation of HT-29 cells was performed either with LPS (50 ng/mL) alone or with LPS and PH-P (0.2–5 mg/mL) or LPS and PH-PT (0.2–5 mg/mL) or LPS and dexamethasone (DEX, 10 or 25 µM). After 3 h, IL-8 secretion levels in the cell culture medium were determined by ELISA. The negative control (NC) represents the incubation of untreated cells. IL-8 (%) is the percentage of IL-8 released by cells relative to its secretion by cells treated only by LPS (PC). All data are represented as the means ± SEM of three experiments performed independently (N = 3). * *p* < 0.05, ** *p* < 0.01, *** *p* < 0.001, **** *p* < 0.0001.

Human HT-29 cell line, an in vitro cell model, was also used to investigate the anti-inflammatory effect of PH-P and PH-PT in both the *S. thermophilus* strains. The cells were co-treated for 3 h with LPS (50 ng/mL) alone or with LPS and various peptide hydrolysate concentrations (0.2, 0.5, 1.0, 3.0, 4.0, and 5.0 mg/mL). Then, ELISA was performed to measure the concentration of pro-inflammatory cytokine IL-8. It was observed that cells treated with LPS (PC) secreted significantly higher IL-8 levels compared to the untreated cells (NC) (*p* < 0.0001; Figure 3). In contrast, the IL-8 secretion levels were significantly reduced by 33.5% and 67.1% when the LPS-induced cells were treated with DEX at 10 µM (*p* < 0.001) and 25 µM (*p* < 0.0001), respectively.

Moreover, cells treated concomitantly with LPS and PH-P or LPS and PH-PT of both *S. thermophilus* strains secreted IL-8 in a concentration-dependent manner (Figure 3). When peptide hydrolysate PH-P or PH-PT obtained from the CNRZ-21N strain was used at concentrations ranging from 0.2 to 3 mg/mL, the secretion of IL-8 levels was not significant (Figure 3B,D). Nevertheless, IL-8 secretion was reduced by 23.2% and 36.1% when PH-P was used at concentrations of 4 mg/mL (*p* < 0.01) and 5 mg/mL (*p* < 0.0001), respectively, compared to cells only stimulated by LPS (PC; Figure 3B). PH-PT obtained from the same strain decreased the secretion of IL-8 in LPS-induced cells by 27.1 and 47.1% at concentrations of 4 mg/mL (*p* < 0.01) and 5 mg/mL (*p* < 0.0001), respectively, compared to PC (Figure 3D).

Concerning the co-treatment of HT-29 cells with LPS and peptide hydrolysates obtained from the LMD-9 strain, again, a concentration-dependent decline in the secretion of IL-8 was observed. As shown in Figure 3A,C, a significant reduction in the secretion of IL-8 was noticed at a concentration of 5 mg/mL (29.1% inhibition, *p* < 0.01) of PH-P, and at concentrations of 3 mg/mL (19.0% inhibition, *p* < 0.05), 4 mg/mL (44.0% inhibition, *p* < 0.0001), and 5 mg/mL (71.3% inhibition, *p* < 0.0001) of PH-PT. Like peptide hydrolysates obtained from the CNRZ-21N strain, no significant difference in the IL-8 decrease was shown at concentrations ranging from 0.2 to 1 mg/mL (Figure 3A,C) by the PH-P and PH-PT obtained from the LMD-9 strain.

Interestingly, no significant difference in the decrease in IL-8 secretion was observed between cells treated with DEX at 10 µM and cells treated with PH-PT at 3 or 4 mg/mL obtained from LMD-9 or cells treated with PH-PT at 4 or 5 mg/mL obtained from CNRZ-21N. Furthermore, the difference in IL-8 secretion was not significant between the cells treated with PH-PT at 5 mg/mL obtained from LMD-9 and cells treated with DEX at 25 µM.

Regarding the cytotoxicity, such an effect was not observed for all of the tested concentrations of LPS, DEX, and peptide hydrolysates (PH-P and PH-PT) obtained from both strains since the overall viability of cells after 3 h of contact with these molecules was greater than 98%.

## 4. Discussion

Chronic inflammation-related diseases such as arthritis or atherosclerosis, type 2 diabetes, inflammatory bowel disease, and other cardiovascular diseases are mainly related to the excessive production of pro-inflammatory mediators [27,28]. One of the effective ways to prevent or delay onset of chronic inflammatory diseases could be the regular consumption of foods containing molecules that may modulate the production of these inflammatory mediators. One such food component is *S. thermophilus*, which belongs to lactic acid bacteria and is extensively employed in the manufacture of fermented dairy products. Hence, this bacterium is regularly consumed as a part of dairy products worldwide. In vitro and in vivo studies revealed that several strains of *S. thermophilus*, even non-living, displayed anti-inflammatory properties [17,19,29]. However, there is little evidence that its surface proteins could contribute to the modulation of inflammation. Our previous study has shown that a peptide hydrolysate resulting from tryptic shaving followed by the trypsinolysis of the surface proteins of *S. thermophilus* LMD-9 displayed a concentration dependent anti-inflammatory activity in THP-1 macrophages and HT-29 cells [24].

During passage through the digestive tract, proteins are known to be widely hydrolyzed by gastrointestinal enzymes, resulting in the release of free amino acids and a great number of peptides [30]. The food bolus during its transit through the GIT encounters the stomach first. It is well-known that stomach acidic conditions can modify the dietary protein structure and subsequently enhance protein degradation [31]. In the stomach, food proteins encounter pepsin, the initial protease of the digestive tract, which hydrolyzes peptide bonds preferably after bulky residues. However, pepsin is less specific as its cleavage specificity does not only depend on the residues of the peptide bond to be cleaved, but is also influenced by the nature of adjacent residues in the n − 4 to n + 4 positions [32]. As hydrolysis by pepsin accounts for about 20% of overall hydrolysis in the GIT [33], subsequent phases of protein hydrolysis occur in the gut through endo- and exopeptidase activities including that of the trypsin. Trypsin is a pancreatic endopeptidase that has narrow specificity to cut the peptide bonds at the C-terminal side of the lysine or arginine residues [34]. In this study, the activity toward the inflammation of hydrolysates obtained by peptic shaving followed or not by trypsinolysis was evaluated using two *S. thermophilus* strains differing in surface proteins and having demonstrated different anti-inflammatory properties. Peptide hydrolysates were recovered from the surface proteins of *S. thermophilus* LMD-9 (PrtS^+^) and CNRZ-21N (PrtS^−^) strains by two different approaches. First, peptic hydrolysates were recovered by shaving the surface of live bacterial cells with pepsin. In a second approach, the polypeptides/peptides generated by the peptic shaving of surface proteins were further hydrolyzed with trypsin.

After surface shaving of the LMD-9 strain with pepsin followed or not by trypsinolysis, the identified peptides overwhelmingly came from surface proteins, mainly the modular protein (2′:3′-cyclic nucleotide 2′-phosphodiesterase) and the PrtS protein, but a few corresponding to cytoplasmic proteins were also highlighted (Table 1 and Table 2). In the case of the surface shaving of the CNRZ-21N strain, although the peptides belonging to the surface proteins were present, the highest number of peptides belonged to two cytoplasmic proteins: phosphocarrier HPr (histidine-containing protein) and the large subunit of the chaperonin GroEL (Table 1 and Table 2). This is due to the fact that the two major peptide generating surface proteins in the LMD-9 strain are absent in the CNRZ-21N strain. Nevertheless, the presence of peptides belonging to cytoplasmic proteins in hydrolysates of both strains could be explained either by slight cell lysis during shaving or the non-classical secretion of cytoplasmic proteins [35]. Indeed, cell lysis, even very minor, might be enough to find peptides from cytoplasmic proteins with those from surface proteins, as the mass spectrometry technique is extremely sensitive to detect peptides of molecules that are present in negligible proportions [35]. In the present study, we did not observe any cell lysis as intracellular lactate dehydrogenase activity was not detected in the supernatants obtained after shaving. Furthermore, it has been reported that cytoplasmic proteins such as moonlighting proteins without any known secretion/exporting signal might have reached the surface by a non-classical secretion pathway that are still unknown [36,37,38]. Mu et al. (2020) demonstrated that the enolase EnoM from *S. thermophilus* could bind to the cell surface [Mu 2020]. On the other hand, GroEL, an intracellular chaperon, could be found in the surface proteomes because it has a transient interaction with the membrane or with proteins associated with the membrane to exert secondary functions [39]. These proteins could anchor to the surface by non-covalent, electrostatic interactions with negatively charged molecules like the teichoic acids of Gram-positive bacteria, because many cytoplasmic proteins (e.g., those ribosomal) have a positive net charge [40,41].

In addition, the difference shown in the proteins identified in the two strains is probably due to the different protein composition of their cell surface. It was already reported that the most representative protein detected after tryptic shaving of the LMD-9 strain was PrtS [24], whereas this protein is lacking in the CNRZ-21N strain. Furthermore, another housekeeping surface protein HtrA, common in both strains, was identified in both strains. However, for a recently reported surface protease SepM, only three peptides were found after peptic shaving of CNRZ-21N [4]. Hence, it can be hypothesized that the identification of more cytoplasmic proteins, rather than surface proteins in the CNRZ-21N strain, might be due to a lower quantity of surface proteins, in particular to the absence of the PrtS protease.

The anti-inflammatory activity of PH-P and PH-PT from the *S. thermophilus* LMD-9 and CNRZ-21N strains was evaluated in vitro in two cell models, i.e. HT-29 cells and THP-1 macrophages. First, it should be noted that PH-P and PH-PT of both strains displayed no cytotoxic effects at all the tested concentrations, irrespective of the cell model used.

Our results showed an increased secretion of the pro-inflammatory cytokines IL-1β, TNF-α, and IL-8 as well as an increased Pro-IL-1β and COX-2 expression in LPS-induced THP-1 macrophages, whereas co-treatment of the THP-1 macrophages with LPS and PH-P and PH-PT at a concentration of 3 mg/mL from the *S. thermophilus* LMD-9 and CNRZ-21N strains demonstrated an anti-inflammatory activity since the production of IL-1β and IL-8 cytokines, and Pro-IL-1β expression were significantly decreased by the peptide hydrolysates. Previously, it has been shown that tryptic peptide hydrolysate recovered from the surface proteins of the LMD-9 strain significantly reduced the pro-inflammatory cytokine expression in LPS-induced THP-1 macrophages [24], and this finding is in agreement with our present results. Likewise, it has been shown that a daily intake of *Lactobacillus acidophilus* DSM 20079T to healthy piglets enhanced the production of IL-10, an anti-inflammatory cytokine, in plasma and decreased the pro-inflammatory response of PBMC. The anti-inflammatory effects of *Lactobacillus acidophilus* DSM 20079T was mainly attributed to the extracellular proteins of this bacterium [42]. Another study showed that peptidoglycan, a major component of the cell wall of *Lactobacillus*, decreased the inflammatory response by upregulating IL-10 production in the colon [43]. Likewise, Kim et al. (2021) showed that surface layer proteins isolated from the probiotic lactic acid bacteria of kefir decreased, in RAW 264.7 macrophages, the secretion of the pro-inflammatory cytokine IL-6 and the production of the protein NF-κB p65 [44].

Furthermore, the effect of peptide hydrolysates of the two strains on the expression of COX-2 was also determined, as COX-2 production is induced in response to inflammatory signals such as cytokines or the bacterial endotoxin LPS [45]. Therefore, the inhibition of COX-2 expression is important in the therapeutic management of inflammatory diseases. The present study demonstrated that PH-P from CNRZ-21N decreased COX-2 expression unlike PH-P from LMD-9, which showed no significant change. Hence, based on a high IL-10/IL-12 ratio, the greater anti-inflammatory activity in the PBMC cell model of the CNRZ-21N strain reported by Junjua et al. (2016) [19] might be due to the fact that the peptide hydrolysates of this strain impacted the secretion/expression of all of the tested cytokines/proteins compared to the LMD-9 strain. The peptide hydrolysate obtained after shaving the surface proteins of the LMD-9 strain with trypsin, followed by trypsinolysis, decreased the expression of COX-2 by 63.6% at a concentration of 3 mg/mL [24]. PH-P obtained from the same strain and tested at the same concentration had no effect on this expression. The active sequence(s) obtained by tryptic shaving followed by trypsinolysis of the surface proteins of the LMD-9 strain could not be released by peptic shaving. Thus, it is speculated that PH-P of the LMD-9 strain did not contain polypeptides/peptides able to reduce COX-2 expression. In contrast, PH-PT obtained from the same strain led to the reduction in COX-2 expression by 63.1%, a percentage close to that found for the tryptic hydrolysate of the surface protein of the LMD-9 strain [24]. This might be explained by the generation of active peptide(s) by the subsequent tryptic hydrolysis of the peptic hydrolysate.

PH-P and PH-PT of both *S. thermophilus* strains were not able to decrease the secretion of TNF-α in LPS-induced THP-1 macrophages, which is consistent with our previous study since the tryptic hydrolysate obtained from surface proteins of the LMD-9 strain had no effect on TNF-α secretion [24].

The anti-inflammatory activity of PH-P and PH-PT obtained from both strains was also assessed in the human colorectal adenocarcinoma HT-29 cell model by quantifying IL-8, one of the predominant pro-inflammatory cytokines secreted by these cells [46]. The LPS-stimulated cells showed significantly reduced IL-8 secretion upon treatment with PH-P or PH-PT from the CNRZ-21N strain at 4 mg/mL and 5 mg/mL. The reduction in IL-8 secretion was only significant at 5 mg/mL after the cells’ treatment with PH-P from the LMD-9 strain. Similarly, HT-29 cells co-treated with LPS and PH-PT from the LMD-9 strain (3–5 mg/mL) showed an anti-inflammatory activity since the production of IL-8 was significantly decreased by this hydrolysate and the reduction was more pronounced at 5 mg/mL, i.e. 71.3%.

Irrespective of the phenotype of the strain (PrtS^+^ or PrtS^−^) and the digestive enzymes used, the proteolytic release of peptides mainly from the surface proteins of the LMD-9 strain and from the surface proteins, and to some extent, the cytoplasmic proteins of the CNRZ-21N strain, led to the production of hydrolysates that were capable of modulating the inflammatory response in vitro. It was observed that tryptic hydrolysis did not impact the anti-inflammatory activity of the sequences already generated by peptic shaving of the CNRZ-21N strain. Nevertheless, it seemed that subsequent tryptic hydrolysis of the polypeptides/peptides obtained after peptic shaving of LMD-9 released anti-inflammatory sequence(s) with a greater efficiency in the reduction in COX-2 expression in the THP-1 macrophages and IL-8 secretion in the HT-29 cells. Indeed, the sequence of peptides released varies depending on the enzyme type and, therefore, its biological activity [47]. Generally, shorter and low-molecular-weight peptides are less susceptible to hydrolysis by digestive enzymes and are absorbed more efficiently than larger peptides [48].

Despite the difference in the number of peptides and the specific sequence distribution of hydrolysates obtained from the two strains LMD-9 and CNRZ-21N, both showed anti-inflammatory activity. Therefore, the absence of the PrtS protease, dominantly present on the surface of the LMD-9 strain, did not impact the capacity of the CNRZ-21N strain to be a source of anti-inflammatory peptides after the action of gastric or gastric and pancreatic proteases.

According to French or American legislation, a product is designated as yogurt if it contains at least 10^7^ live bacteria per mL. *S. thermophilus* represents about 50% of the total bacterial population of yogurt and commercially available yogurts can contain up to 10^10^ CFU/mL of this bacterium in the finished product [49]. According to the previously used calculation method based on *E. coli* composition [24], a serving size of 125 g yogurt could contain up to 1.25 × 10^12^ CFU of *S. thermophilus*, leading to a total amount of about 150 mg of proteins. Consequently, the consumption of a single serving size of 125 g yogurt may provide enough peptides after the digestion of these proteins to exert an anti-inflammatory effect in the consumer.

Our findings demonstrated that two phenotypically different *S. thermophilus* strains, LMD-9 (PrtS^+^) and CNRZ-21N (PrtS^−^), with different anti-inflammatory properties, released peptides after peptic shaving, followed or not by a tryptic hydrolysis, which exhibited anti-inflammatory action in the THP-1 macrophages as well as in the HT-29 cells induced by LPS through the modulation of pro-inflammatory mediators. Thus, peptides liberated from some *S. thermophilus* strains in the gastrointestinal tract during the digestion of a product containing this bacterium may exert anti-inflammatory effects and reduce the risk of inflammation-related chronic pathologies.

## Figures and Tables

**Table 1:** Identification of proteins via LC-MS/MS analysis of peptides obtained after peptic shaving of *S. thermophilus* LMD-9 and CNRZ-21N.

					LMD-9		CNRZ-21N	
Protein ID	New Locus Number	Description	MW (kDa)	Location	Nb Specific Sequences Identified	Coverage (%)	Nb Specific Sequences Identified	Coverage (%)
**Nucleotide metabolism and transport**								
STER_0198|ID:1898877|	STER_RS00970	2′:3′-cyclic-nucleotide 2′-phosphodiesterase (modular protein)	91.21	CS	205	75.44		
STER_1118|ID:1899510|fliY|	STER_RS05530	Cystine transporter subunit; periplasmic-binding component of ABC superfamily	30.94	Cyto	13	42.09		
**Post-translational modification, protein turnover and chaperone function**										
STER_0846|ID:1899371|	STER_RS04165	Exported protein of unknown function (subtilisin-like serine protease PrtS)	173.05	CS	343	74.80		
STER_0253|ID:1898921|groL|	STER_RS01230	Cpn60 chaperonin GroEL, large subunit of GroESL	56.89	Cyto	14	22.78	17	31.11
STER_2002|ID:1899687|degP|	STER_RS09790	Serine endoprotease (protease Do), membrane-associated	42.77	CM/M			14	30.10
**Translation**								
STER_0524|ID:1899138|tufB|	STER_RS02570	Protein chain elongation factor EF-Tu (duplicate of tufA)	43.84	Cyto	14	35.34		
STER_1904|ID:1900567|rplB|	STER_RS09330	50S ribosomal subunit protein L2	29.91	Cyto			8	29.50
STER_0568|ID:1899173|rplL|	STER_RS02800	50S ribosomal subunit protein L7/L12	12.35	Cyto	21	61.79		
STER_1936|ID:1899661|	STER_RS09470	50S ribosomal protein L28	6.91	Cyto	18	96.83	8	61.91
STER_1899|ID:1900562|rpmC|	STER_RS09305	50S ribosomal subunit protein L29	7.90	Cyto	12	68.12		
STER_1889|ID:1900552|rpmD|	STER_RS09255	50S ribosomal subunit protein L30	6.39	Cyto	51	88.53	9	44.26
STER_1953|ID:1899670|rpmF|	STER_RS09555	50S ribosomal subunit protein L32	6.78	Cyto	12	83.61		
STER_1954|ID:1899671|rpmG|	STER_RS09560	50S ribosomal subunit protein L33	5.92	Cyto	19	92.00		
STER_1894|ID:1900557|	STER_RS09280	30S ribosomal protein S14 type Z (BS21)	7.07	Cyto	11	82.26		
STER_0850|ID:1899831|rpsT|	STER_RS04185	30S ribosomal subunit protein S20	8.40	Cyto	10	82.28	10	89.87
STHERMOCNRZ21N_v1_31103|ID:59661243|rplX|		Ribosomal protein L24 (BL23)	10.86	Cyto			16	69.61
**Carbohydrate metabolism and transport**								
STER_0684|ID:1899266|eno|	STER_RS03365	Enolase	46.95	CS/M/Cyto	10	22.30	8	19.08
STER_1243|ID:1900052|	STER_RS06135	Phosphocarrier protein HPr (histidine-containing protein)	8.91	Cyto	35	98.86	17	98.86
STER_1876|ID:1900541|kbaY|	STER_RS09185	Tagatose 6-phosphate aldolase 1, kbaY subunit	31.51	Cyto	10	31.63	8	30.27
**Cell wall/membrane/envelope biogenesis**								
STER_0042|ID:1898743|	STER_RS00210	Secreted 45 kDa protein precursor	46.45	CS	12	28.73		
**Amino acid transport and metabolism**								
STER_1411|ID:1900180|	STER_RS06940	Putative transporter subunit: periplasmic-binding component of ABC superfamily	72.18	CS	14	45.59		
STHERMOCNRZ21N_v1_30588|ID:59660728|amiA|		Oligopeptide-binding protein AmiA	71.92	CS/M			12	35.98
STHERMOCNRZ21N_v1_30299|ID:59660439|		Polar amino acid ABC uptake transporter substrate binding protein	30.99	CS			8	32.73
STER_0340|ID:1898987|metQ|	STER_RS01655	DL-methionine transporter subunit; periplasmic-binding component of ABC superfamily	3.89	M			10	33.22
**Transport**								
STER_1539|ID:1900277|	STER_RS07565	Glutamine-binding protein precursor (GlnBP)	31.30	CS	17	44.91		
STHERMOCNRZ21N_v1_10277|ID:59659427|nupN|		Lipoprotein involved in guanosine transport	37.64	CS			12	33.33
**Energy production and conversion**								
STER_0519|ID:1899133|atpA|	STER_RS02545	F1 sector of membrane-bound ATP synthase, alpha subunit	54.54	CM			14	27.49
STER_0521|ID:1899135|atpD|	STER_RS02555	F1 sector of membrane-bound ATP synthase, beta subunit	50.84	CM			10	24.73
**Unknown or other function**								
STER_1963|ID:1899676|	STER_RS09605	Conserved protein of unknown function (CsbD family protein)	7.08	Cyto	49	97.06		
STER_1141|ID:1899514|	STER_RS10465	Exported protein of unknown function	6.26	_	12	71.19		
STER_0856|ID:1899377|	STER_RS04220	CD4+ T-cell-stimulating antigen precursor	37.62	CS	25	55.18	12	33.33
STER_0576|ID:1899177|	STER_RS02840	Mucus binding protein precursor (fragment)	108.40	CS	58	56.17		
STER_0478|ID:1899094|	STER_RS02350	Exported protein of unknown function	50.50	CS	21	63.00		
STER_0378|ID:1899011|	STER_RS01835	Membrane-bound protein LytR (modular protein)	43.94	Cyto	18	34.23		
STER_0715|ID:1899786|		Protein of unknown function	4.92	_	10	81.82		
STHERMOCNRZ21N_v1_10861|ID:59660011|mapZ|		Mid-cell-anchored protein Z	66.10	CM/M			8	15.46

CS: cell surface protein; M: membrane located protein; CM: cell membrane located protein; Cyto: cytoplasmic protein.

**Table 2:** Identification of proteins via LC-MS/MS analysis of peptides obtained after peptic shaving followed by trypsinolysis of *S. thermophilus* LMD-9 and CNRZ-21N.

					LMD-9		CNRZ-21	
Protein Id	New Locus Number	Description	MW (kDa)	Location	Nb Specific Sequences Identified	Coverage (%)	Nb Specific Sequences Identified	Coverage (%)
**Post-translational modification, protein turnover, chaperone function**								
STER_0846|ID:1899371|	STER_RS04165	Exported protein of unknown function (subtilisin-like serine protease PrtS)	173.05	CS	190	62.26		
STER_2002|ID:1899687|degP|	STER_RS09790	Serine endoprotease (protease Do), membrane-associated	42.77	CM/M			19	40.05
STER_0253|ID:1898921|groL|	STER_RS01230	Cpn60 chaperonin GroEL, large subunit of GroESL	56.89	Cyto	9	17.59	18	34.44
STER_0648|ID:1899776|clpA|	STER_RS03195	ATPase and specificity subunit of ClpA-ClpP ATP-dependent serine protease, chaperone activity	83.69	_	9	5.98		
STER_0163|ID:1898844|dnaK|	STER_RS00790	Chaperone Hsp70, co-chaperone with DnaJ	64.76	Cyto	10	10.86	8	19.57
**Nucleotide metabolism and transport**								
STER_0198|ID:1898877|	STER_RS00970	2′:3′-cyclic-nucleotide 2′-phosphodiesterase (modular protein)	91.21	CS	152	59.15		
**Cell wall/membrane/envelope biogenesis**								
STER_0042|ID:1898743|	STER_RS00210	Secreted 45 kDa protein precursor	46.45	CM	25	46.05		
**Energy production and conversion**								
STER_0519|ID:1899133|atpA|	STER_RS02545	F1 sector of membrane-bound ATP synthase, alpha subunit	54.54	CM	14	27.49	11	24.10
STER_0521|ID:1899135|atpD|	STER_RS02555	F1 sector of membrane-bound ATP synthase, beta subunit	50.84	CM	10	24.73	13	34.76
**Transport**								
STER_0340|ID:1898987|metQ|	STER_RS01655	DL-methionine transporter subunit; periplasmic-binding component of ABC superfamily	32.89	M	10	33.22	9	32.23
STHERMOCNRZ21N_v1_10277|ID:59659427|nupN|		Lipoprotein involved in guanosine transport	37.64	CS	12	33.33	14	40.62
STHERMOCNRZ21N_v1_30299|ID:59660439|		Polar amino acid ABC uptake transporter substrate binding protein	30.99	CS	8	32.73		
STHERMOCNRZ21N_v1_30588|ID:59660728|amiA|		Oligopeptide-binding protein AmiA	71.92	CS			14	43.29
STHERMOCNRZ21N_v1_10753|ID:59659903|livJ|		Branched-chain amino acid ABC uptake transporter substrate-binding protein	41.81	CS/M			8	23.98
**Translation**								
STER_1889|ID:1900552|rpmD|	STER_RS09255	50S ribosomal subunit protein L30	6.39	Cyto	9	44.26	10	57.38
STHERMOCNRZ21N_v1_31096|ID:59661236|rpmD|		Ribosomal protein L30 (BL27)	6.39	Cyto	18	81.97		
STER_0639|ID:1899231|	STER_RS03135	40S ribosomal protein S1	43.88	Cyto	8	7.48		
STER_1904|ID:1900567|rplB|	STER_RS09330	50S ribosomal subunit protein L2	29.91	Cyto	8	29.5	9	36.33
STER_0568|ID:1899173|rplL|	STER_RS02800	50S ribosomal subunit protein L7/L12	12.35	Cyto	8	59.35	11	48.78
STER_0850|ID:1899831|rpsT|	STER_RS04185	30S ribosomal subunit protein S20	8.40	Cyto	10	89.87	9	89.87
STER_1905|ID:1900568|rplW|	STER_RS09335	50S ribosomal subunit protein L23	10.78	Cyto			8	45.46
STHERMOCNRZ21N_v1_31103|ID:59661243|rplX|		Ribosomal protein L24 (BL23)	10.86	Cyto	16	69.61	21	80.39
**Carbohydrate metabolism and transport**								
STER_1243|ID:1900052|	STER_RS06135	Phosphocarrier protein HPr (Histidine-containing protein)	8.91	Cyto	11	60.23	19	98.86
STER_0684|ID:1899266|eno|	STER_RS03365	Enolase	46.95	CS/M/Cyto			9	26.44
**Unknown or other function**								
STER_0856|ID:1899377|	STER_RS04220	CD4+ T-cell-stimulating antigen precursor	37.62	CS			14	40.62
STER_0576|ID:1899177|	STER_RS02840	Mucus binding protein precursor (fragment)	108.4	CS	66	45.24		
STER_0478|ID:1899094|		Exported protein of unknown function	50.50	_	14	42.00		
STER_1317|ID:1899536|		Conserved protein of unknown function	6.81	_			11	80.00
STHERMOCNRZ21N_v1_10861|ID:59660011|mapZ|		Mid-cell-anchored protein Z	66.10	CM/M	8	15.46		

CS: cell surface protein; M: membrane located protein; CM: cell membrane located protein; Cyto: cytoplasmic protein.

## Data Availability

Data are contained within the article.
